# TNF-α exacerbates postoperative plantar pain by regulating the expression of Nav1.8

**DOI:** 10.1371/journal.pone.0351249

**Published:** 2026-07-17

**Authors:** Zilu Shang, Yu Li, Yun Zhang, Yajuan Gong

**Affiliations:** 1 The First Hospital of Anhui University of Science and Technology, Huainan, China; 2 College of Integrated Traditional Chinese and Western Medicine, Hebei Medical-University, China; 3 Department of Clinical-Laboratory, Shaanxi Provincial-People’s Hospital, Xi’an. Shaanxi, China; University of Minho, PORTUGAL

## Abstract

Postoperative pain (POP) is one of the most common complications of surgical procedures. Using a rat plantar incision model, we investigated the interactions between tumor necrosis factor-α (TNF-α), nuclear factor-κB (NF-κB), and the voltage-gated sodium channel Nav1.8, to uncover the neural basis of POP. Our research demonstrates that, within the dorsal root ganglion (DRG), TNF-α enhances pain behaviors by driving NF-κB–dependent overexpression of Nav1.8 in neurons, further elucidating the molecular basis of POP. This study identifies the TNF-α/NF-κB/Nav1.8 axis as a critical pathway for therapeutic intervention, thereby establishing a theoretical foundation for targeting cytokine signaling to alleviate POP.

## Introduction

Postoperative pain (POP) is a typical complication of surgical procedures, not only prolonging recovery time but also significantly reducing patients’ quality of life. Studies have shown that suboptimal management of POP has the potential to result in adverse outcomes including functional disability, reduced capacity for daily living, and the onset of chronic pain [1,2].

It is widely acknowledged that POP is closely linked to acute inflammatory responses, which are essential for tissue repair. However, neuroinflammatory responses can sensitize peripheral pain receptors, resulting in persistent pain [3,4]. It is vital to ensure effective management of acute POP to avoid its progression to chronic pain. A substantial body of research has identified the severity and duration in the acute pain phase as key predictors of the development of chronic postsurgical pain (CPSP) [5,6]. During surgical procedures, nerve damage and inflammatory responses can trigger hypersensitivity in both peripheral and central pain pathways, which is considered a crucial element in the evolution of chronic pain [3,4]. Furthermore, the occurrence of postoperative complications is related to an increased risk of persistent pain [7].

To effectively manage POP, preventive analgesia strategies are widely adopted. These strategies encompass multimodal pain management before and after surgery, aimed at reducing pain severity and duration [8,9]. Evidence suggests that local and regional anesthesia techniques can significantly lower the incidence of chronic pain following certain procedures [10]. Furthermore, personalized POP management approaches are recognized as effective in improving patients’ recovery outcomes and quality of life [11].

The voltage-gated sodium channel Nav1.8, which is encoded by the *SCN10A* gene (Nav1.8 gene), is predominantly characterised by its expression in small-diameter sensory neurons, and it has been demonstrated to play a pivotal role in action potential propagation and in the context of inflammatory pain. This channel is of critical importance in the transmission of pain signals, particularly in the context of inflammatory or neuropathic pain conditions. Comprehension of the expression and functional regulation of Nav1.8 is imperative for the elucidation of its function in pain reception.

Nav1.8 is highly concentrated in small-diameter sensory neurons, which are primarily located in the DRG and cranial ganglia [12]. This specific expression makes Nav1.8 a crucial mediator of pain signal transmission. Studies have shown that the functional regulation of Nav1.8 is associated with interactions between its auxiliary β subunits, where the β3 subunit can reduce the maximum sodium current amplitude of Nav1.8 and delay its recovery from deactivation [13]. Additionally, Nav1.8 accumulates in peripheral nerves during inflammation and nerve injury, a phenomenon linked to the anterograde transport function of KIF5B protein [14]. Although traditionally recognized for its specific expression in small-diameter neurons, recent studies demonstrate that Nav1.8 is also present in large-diameter sensory neurons, indicating functional variations across diverse cell types [15]. In inflammatory pain conditions, the expression levels, and functional regulation, of Nav1.8 are particularly significant. Studies have shown that upregulation of this channel correlates with mechanical hyperalgesia, which may be mediated through protein kinase Cε-induced phosphorylation [16]. Furthermore, the enhanced activity of Nav1.8 has been linked to scorpion venom-induced pain behaviors, further supporting its crucial role in nociception [17]. In neuropathic pain, the expression changes of Nav1.8 are equally significant. Studies indicate that Nav1.8 channel mRNA levels increase in damaged nerves following sciatic nerve injury, while showing no significant changes in the DRG. This suggests its potential role in axonal mRNA transport following neural injury [18]. Furthermore, an increase in the activity of Nav1.8 is essential for the onset of chronic inflammatory pain, and its elevated presence in Aβ fibers might lead to mechanical hyperalgesia. [19].

In summary, the central contribution of Nav1.8 in nociceptive transmission is demonstrated not only through its specific expression in small-diameter sensory neurons, but also through its functional regulation in inflammatory and neuropathic pain. Through in-depth research on Nav1.8, we can better understand its role in pain perception and identify potential targets for developing new analgesic drugs [20,21]. However, the precise mechanism by which TNF-α regulates Nav1.8 in POP remains unclear. Therefore, this study aims to investigate whether TNF-α drives Nav1.8 expression via the NF-κB pathway. We hypothesize that the activation of the TNF-α/NF-κB/Nav1.8 axis in DRG neurons is a key mechanism underlying postoperative hypersensitivity, particularly regarding thermal and spontaneous pain.

## Materials and methods

### Animals

Male Sprague-Dawley (SD) rats weighing approximately 220 g were housed in the Specific Pathogen-Free (SPF) Animal Laboratory of Xi’an Jiaotong University. All rats were kept in an environment that was controlled for temperature and humidity under a 12-hour light/dark cycle. The temperature was maintained at 23°C ± 2°C, and the humidity at 50–60%. The environment was kept quiet and free of odors. Experiments were conducted after one week of acclimatization. This study was conducted in strict accordance with the Guide for the Care and Use of Laboratory Animals and the institutional guidelines of Xi’an Jiaotong University.

### Animal welfare, humane endpoints, and study design details

All procedures were approved by the IACUC of Xi’an Jiaotong University (No. XJTUAE2025−730) and complied with institutional and national guidelines. Humane endpoints were predefined: ≥ 20% body weight loss from baseline (pre-surgery), severe lethargy/unresponsiveness, inability to eat/drink, or severe pain unresponsive to analgesia (assessed by persistent hunching, lack of grooming, or self-mutilation despite treatment); animals meeting criteria would be euthanized within 2 h. Animal health and behavior were monitored at least once daily. Postoperative analgesia was provided with carprofen (5 mg/kg, s.c.) at surgical closure and once daily for the next 3 days. No animals required early euthanasia. All 31 male SD rats survived to the planned endpoint (day 21) and were euthanized by cervical dislocation under deep isoflurane anesthesia after the final behavioral assessment. Personnel were trained in animal handling and ethical procedures.

### Experimental design

Rats were acclimated to the animal facility for 7 days prior to any procedures. Animals were randomly assigned to either the control (non-incisional) group (Con) or the POP (plantar incision) group. The POP model was established as described below. Behavioral assessments, including the mechanical withdrawal threshold (MWT), hot plate test (HPT), and Rotarod test, were performed at baseline (Day 0, pre-surgery) and at multiple post-operative time points: 6 hours, 1 day, 3 days, 5 days, and 7 days. For mechanistic studies involving the NF-κB inhibitor, BAY 11–7082 was administered intrathecally (i.t.) 30 minutes before surgery. Following the final behavioral assessment at designated time points (primarily at the 6-hour peak for molecular assays), rats were euthanized under deep anesthesia, and L4–L5 DRG and spinal cord tissues were harvested for qPCR, Western blot, ELISA, and electrophysiological recordings.

### POP model

A plantar incisional pain model was established in rats. A 1-cm linear incision was made on the plantar surface, commencing 0.5 cm proximal to the toes. After incising the skin, the plantar muscles were gently lifted with fine forceps and longitudinally incised, while preserving the origin, insertion, and attachments of the muscles. Hemostasis was achieved by gentle compression, followed by skin suturing. The control-operated rats underwent the same anesthesia and handling procedures as the POP group, but without the plantar incision. To ensure pharmacological consistency between groups, control animals also received the same analgesic treatment (carprofen, 5 mg/kg, s.c., for 3 days).

### Behavioral tests

#### Open field test (OFT).

Rats were positioned within an open field arena, which measured 40 cm × 40 cm × 25 cm. The software for analyzing animal behaviors recorded the distance traveled in the central region in the core and surrounding areas, plus the overall distance covered over 5 minutes. Before all behavioral tests involving rats, the open field arena received a thorough 75% ethanol cleaning to eliminate odors. To establish a reference for locomotor and exploratory activity, baseline measurements were conducted 24 hours prior to surgery.

#### Hot plate test (HPT).

The stimulated hind paw’s underside contacted the thermal analgesimeter surface (maintained at 55 ± 1 °C) until paw withdrawal reflex occurred. Baseline measurements were obtained one day before the surgical procedure to establish the normal thermal threshold. A digital timer was deployed to gauge the withdrawal latency (in seconds) between paw activation and the subsequent behavioral reaction. Only a distinct unilateral paw withdrawal was recorded as a nocifensive response. During the observation, the stimulated hind paw’s plantar surface was placed on the hot plate, whereas the contralateral hind paw was rested on filter paper (removed from the heated plate) to avoid simultaneous thermal stimulation of both hind paws. Each stimulation was separated by 1 minute, and two alternate evaluations were performed for each hind paw. To prevent tissue injury, testing was limited to 50 seconds. The mean value of the two measurements was calculated and recorded as the baseline (Day 0) for each animal.

#### Von Frey test (VF).

One day prior to surgery, baseline mechanical thresholds were collected. Animals were habituated over a 2-hour period in transparent acrylic chambers (7.5 cm wide × 7.5 cm long × 15 cm high) placed on a raised wire-mesh support platform, which allowed access to the hind paw plantar aspect. Von Frey stimuli were delivered adjacent to the incision site on the plantar surface. Calibrated Von Frey monofilaments (log series, with forces ranging from 0.02 to 1.4 g) were used following the up-down method, beginning with a 0.6-gram filament. Each stimulus was tested twice, with each application lasting 2–3 seconds and separated by ≥30 s to reduce hypersensitivity to mechanical stimuli. A positive reaction was characterized by prompt licking/biting, paw flinching, or brisk withdrawal. The average of these duplicate tests was used for both baseline and post-operative data analysis.

#### Rotarod test.

A rotarod device equipped with a grid-like plastic rod (5-cm-diameter) was employed to assess locomotor coordination. Rats underwent three days of training to reach a stable level of performance. During the assessment phase, a rat was positioned on the rod set to a constant speed of 5 revolutions per minute (rpm), and the latency to fall was recorded. Each rat underwent three training sessions per day, with each trial limited to a cutoff time of 300 s. This training was conducted consecutively for three days. The mean latency to fall on the third day of training was recorded as the baseline for motor coordination. After POP induction, the same formal experimental test was performed.

#### Spontaneous pain behavior assessment.

Baseline spontaneous pain behaviors were recorded one day before surgery to ensure the absence of pre-existing nocifensive responses. Rats were placed in an empty chamber, and the number of times they licked the surgical plantar surface within 5 minutes was recorded.

### Bioinformatics analysis

The gene expression levels in DRG of the plantar incision model at 0 days, 6 hours, 2 days, and 10 days post-surgery were retrieved from the Gene Expression Omnibus (GEO) database (GSE267799), which included 4 samples per group.

Differential expression analysis was carried out using DESeq2[Moderated estimation of fold change and dispersion for RNA-seq data with DESeq2]. Genes with adjusted *P* value < 0.05 and |log_2_(fold change)| > 1 were considered differentially expressed. We used the R package “clusterProfiler”[clusterProfiler 4.0: A universal enrichment tool for interpreting omics data][clusterProfiler: an R package for comparing biological themes among gene clusters], “org.Rn.e.g.,db”, “enrichplot”[enrichplot: Visualization of Functional Enrichment] and “ggplot2”[ggplot2: Elegant Graphics for Data Analysis] to conduct gene ontology (GO)[The Gene Ontology Resource: 20 years and still GOing strong] and the Kyoto Encyclopedia of Genes and Genomes (KEGG)[KEGG for linking genomes to life and the environment] enrichment. Next, we used the R package “qlcMatrix”[qlcMatrix: Utility Sparse Matrix Functions for Quantitative Language Comparison], “igraph”[The igraph software package for complex network research][igraph: Network Analysis and Visualization in R], “ClusterGVis”[ClusterGVis: One-Step to Cluster and Visualize Gene Expression Data] and “ComplexHeatmap”[Complex heatmaps enable the visualization of underlying patterns and inter-variable relationships within high-dimensional genomic datasets.][Integrated Heatmap-Based Data Representation] to generate visualizations and integrate the results.

### Elisa

Blood samples were collected at 6 h post-surgeryand centrifuged at 3000 rpm for 15 minutes to obtain serum. Standard solutions were prepared by serial dilution in Eppendorf tubes and mixed thoroughly(TNF-α ELISA Kit, JL10208-96T, JonlnBio). Blank wells and sample wells were set on the enzyme-linked immunosorbent plate, with 3 replicates per group, followed by sample loading. The test plate was sealed with a special sealing film and maintained at 37°C for one hour. Washing buffer was prepared from concentrated washing solution, following the instructions provided by the manufacturer. The plate was thoroughly washed 3 times (add washing buffer → discard after 2 minutes → pat dry, ensuring no air bubbles remained in the wells). Working Solution B (avidin-conjugated HRP) was prepared and applied to all wells. The dish was resealed with a protective film and reincubated at 37 °C for 30 min, then washed 3 times again (add washing buffer → discard after 2 minutes → pat dry). Upon addition of TMB substrate, the plate was covered with a sealing film and placed in a 37°C incubator shielded from light. The reaction was allowed to proceed for up to 10 minutes and was monitored until a distinct blue color appeared in the wells. Stop solution was added to terminate the reaction, resulting in a color change from blue to yellow. Absorbance readings were acquired 15 minutes after the addition of the stop solutions. At this time, the optical density (OD) was recorded at 450 nm using a microplate reader. A standard curve was generated in Excel (valid when R^2^ > 0.99) to calculate the concentrations of target substances (IL-1β, IL-6, and TNF-α) in the serum samples (IL-1β ELISA Kit, JL13662-96T; IL-6 ELISA Kit, JL14113-96T, JonlnBio).

### qPCR

Total RNA was isolated at 6 h post-surgery using TRIzol® (Thermo Fisher Scientific) from the L4-L6 DRG, and the purity and concentration were verified via NanoDrop 2000 (A260/A280 = 1.8–2.0). First-strand cDNA was synthesised using the PrimeScript™ RT Reagent Kit (TaKaRa) (20 μL system: 4 μL 5 × buffer, 1 μL RT enzyme mix, 1 μL gDNA eraser, 1 μg RNA, nuclease-free water to final volume, reaction: 42°C for 2 min, 37°C for 15 min, 85°C for 5 s). qPCR was carried out in a 20 μL reaction mixture (10 μL SYBR® Premix Ex Taq™ II (TaKaRa), 0.4 μL of each primer (forward and reverse, 10 μM, the sequences are listed in S1 File), 2 μL cDNA, 7.2 μL nuclease-free water) using a StepOnePlus™ real-time PCR instrument (Applied Biosystems, Thermo Fisher Scientific) under the following thermal cycling conditions: 95°C for 30 s, followed by 40 cycles of 95°C for 5 s and 60°C for 30 s, a melting curve analysis (95°C for 15 s, 60°C for 1 min, 95°C for 15 s) was conducted to confirm amplification specificity. Relative mRNA levels were quantified using the 2^(-ΔΔCt) method, normalized to GAPDH as an endogenous control, and each sample was run in triplicate.

### Western blotting

Proteins were extracted from L4–L6 DRG at indicated time points, and the concentration of these proteins was measured using a BCA assay kit. An equal quantity of protein (50 μg per lane) was used for electrophoresis. Samples were resolved by sodium dodecyl sulfate-polyacrylamide gel electrophoresis (SDS-PAGE) and then electrotransferred to polyvinylidene difluoride (PVDF) membranes. The PVDF membranes were blocked in 5% nonfat dry milk for 2 hours at ambient temperature, followed by overnight incubation period at 4°C with primary antibodies specific for TNF-α (A22227, Abconal, 1:1500), IL-6 (YP-Ab-16022, UpingBio,1:1500), IL-1β (YP-Ab-16004, UpingBio, 1:1500), Nav1.8 (DF13241, Affinity), and β-actin (GB11001, Servicebio). After washing, the membrane was probed with diluted secondary antibodies (GB23303, GB23301, Servicebio, 1:5000) for 90 min at ambient temperature. Immunoblots were visualized using a highly sensitive ECL chemiluminescence detection system.

### Acute cell isolation

Adult SD rats received isoflurane anesthesia followed by euthanasia via decapitation. The dorsal skin and muscles were incised, and the spine was exposed to isolate DRG from the lumbar L4 to lumbar L6 segments. DRG were carefully collected, their nerve roots were trimmed, and the tissues were placed in 3 mL of sterile DMEM (without bicarbonate and serum) supplemented with 3.125 mg/mL neutral protease and 5 mg/mL collagenase type I. Samples underwent digestion at 37°C with continuous agitation over 45 minutes. After digestion, DRG tissues were gently centrifuged, followed by discarding the supernatant and collecting the precipitated DRG neurons. Neurons were rinsed with high-glucose DMEM enriched with 10% FBS, penicillin (100 U/mL), and streptomycin (100 μg/mL) to prepare a single-cell suspension. The samples were plated on poly-D-lysine and laminin-coated glass slides. A 20 μL aliquot of the cell suspension was dropped onto the center of each coverslip, which was then placed in a humidified 37°C, 5% CO_2_ incubator for 30 min to allow neuron attachment. Subsequently, 980 μL of DMEM was gently added to the edge of each well (total volume per well: 1 mL) to prevent detachment of weakly adherent cells. Isolated DRG neurons were used within 6 hours of culture.

### Whole-cell patch-clamp recording

#### Cell preparation and recording conditions.

Male SD rats (6–8 weeks old) were selected for the model. Acutely dissociated L4–L6 DRG neurons were isolated and digested in a 37°C water bath for 45 min. The isolated cells were plated into multi-well plates and allowed to attach for 6 hours before recording. Based on classical size-based classification, we specifically targeted small-diameter DRG neurons (diameter ≤ 27 μm) under the microscope. These small-diameter neurons are generally considered to be C-fiber nociceptors, which are closely associated with pain generation. A total of 3–5 cells per group were recorded, obtained from 3 animals. Whole-cell patch-clamp recordings were performed at room temperature (23–25°C) using an EPC10 amplifier and Igor Pro software (HEKA Instruments, Germany). Recording pipettes were fabricated from borosilicate glass capillaries using a P-97 puller (Sutter Instrument) and had a tip resistance of 3–5 MΩ when filled with the intracellular solution. For mechanistic studies, neurons were pre-treated with 10 mM BAY 11–7082 (HY-1873, MCE) added directly to the medium for 3 hours prior to recording.

#### Solutions and pharmacological isolation.

The intracellular solution contained (in mM): 130 K-gluconate, 6 NaCl, 11 EGTA, 10 HEPES, 1 CaCl_2_, 4 NaOH, 1 MgCl_2_·6H_2_O, 2 MgATP, and 0.2 Na_2_GTP (pH 7.2). The extracellular solution consisted of (in mM): 124 NaCl, 26 NaHCO_3_, 10 Glucose, 4.5 KCl, 1.2 NaH_2_PO_4_, 1 MgCl_2_, and 2 CaCl_2_ (pH 7.4). To separate potassium ions and calcium ions, TEA-Cl, CsCl, CdCl_2_, and 4-AP need to be added to the external solution, and CsCl needs to be added to the internal solution. All chemical reagents were sourced from Sigma-Aldrich. To specifically isolate Nav1.8-mediated currents, 100 μM Suzetrigine (VX548; HY-148800, MCE) was utilized. The Nav1.8 component was defined as the VX548-sensitive current, calculated by digital subtraction of the residual current after drug application from the total sodium current.

#### Recording protocols and quality control.

Voltage-gated sodium currents were recorded in voltage-clamp mode. DRG neurons were held at a holding potential of –80 mV. Currents were elicited using a step protocol, progressively increasing the test voltage from –80 mV to +50 mV in 10-mV increments, with a pulse duration of 200 ms (as depicted in Fig 5J).

Action potentials (APs) were recorded in current-clamp (I-clamp) mode. With the holding current maintained at 0 pA, a 100-pA depolarizing current was injected into the cell for a duration of 1 s.

Data acquisition was performed at a sampling frequency of 2 kHz for APs and 10 kHz for sodium currents, with >80% series resistance compensation and low-pass filtering. Only cells with a seal resistance >1 GΩ and an initial series resistance <35 MΩ were analyzed; recordings were discarded if the series resistance shifted by >15% during the experiment.

### Intrathecal injection

A laminar flow hood was disinfected with 75% ethanol. Rats were positioned in a prone position, secured to the surgical plate by grasping the iliac bones with the non-dominant hand, and the upper body was covered with sterile gauze or a towel to calm the animal. Tension was maintained on the dorsal skin using the thumbs and index fingers. The skin at the injection site was shaved and disinfected. The dominant hand was used to palpate the midline of the vertebral space between the bilateral iliac bones, and the L5–L6 intervertebral space was identified as the injection site. The base of the tail was slightly rotated to identify the midline of the spinal column. The bevel of a 30-gauge needle was adjusted to face the animal’s head before injection. The animal was firmly fixed in place and the 25 μL Hamilton micro-syringe was aligned with the midline of its spine. Starting at a 70–80° angle, the needle was inserted into the vertebral midline dimple. Once bone contact was detected, the angle was lowered to ~30°, and the needle was guided into the intervertebral space. A distinct sudden tail flick was considered a sign of successful insertion into the intrathecal space. To ensure a precise delivery of BAY 11–7082 (40 μg in 10 μL saline, B5556, Sigma) or recombinant TNF-α protein (GF314, Sigma), the micro-syringe was mounted on a micro-infusion pump (RWD, China), and the injection was performed 30 min before POP induction. The injection was performed at a rate of 1 μL every 4 seconds. After injection, the needle was maintained at the injection site for 2 min, then gently rotated and withdrawn to prevent fluid leakage.

### Data analysis and statistics

All experiments and data analyses were performed blinded to the experimental group assignments. Replicates (n) in figures denote the count of individual animals per condition. Data analysis was conducted using GraphPad Prism version 10. Analytical procedures employed two-way ANOVA as dictated by the study’s design, as detailed in respective figure captions. Non-significant results are indicated as “n.s.” Results are expressed as mean ± SEM.

## Results

### Behavioral assessments of pain sensitivity and locomotor function following surgery

**Fig 1:**
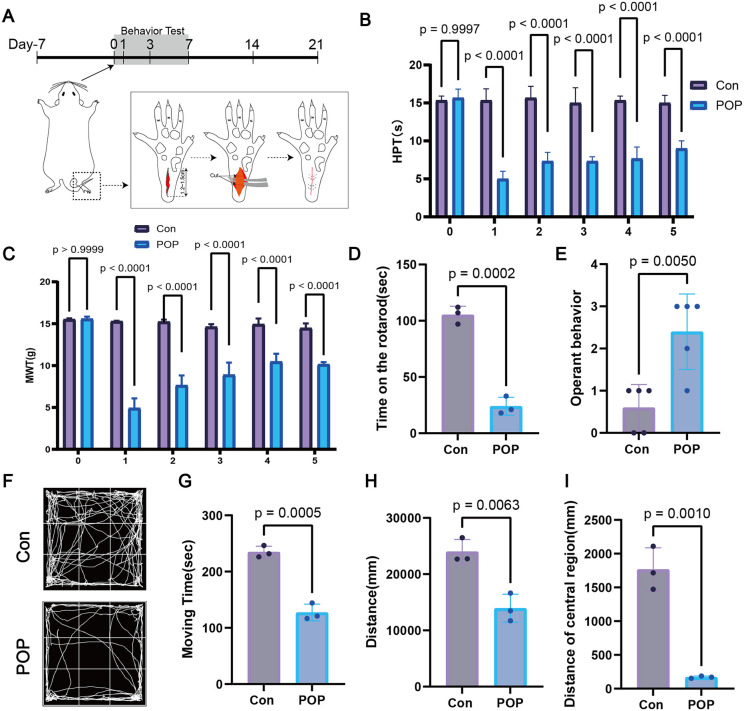
Behavioral assessments of motor function and pain sensitivity following surgery. (A) Timeline of behavioral tests conducted at baseline (Day −1) and post-surgery on indicated days (represented as 0-5 in B-C). (B) Hind paw withdrawal latency (HPT, seconds) measured at each time point. (C) Mechanical withdrawal threshold (MWT, grams) at each time point. (D) Rotarod performance measured as time spent on the rotating rod (seconds). (E) Spontaneous nocifensive behavior quantified as the number of licking events (operant behavior) recorded in an empty chamber. (F) Representative locomotor traces from the open field test. (G) Total moving time (seconds) in the open field test. (H) Total distance traveled (mm) in the open field test. (I) Distance traveled specifically in the central region of the open field (mm). Data were analyzed using two-way ANOVA. n = 3-5 per group. Non-significant results were indicated as “ns”. Data were expressed as mean ± SEM. *P* < 0.05 was considered statistically significant.

The successful establishment of the POP model was confirmed through a series of behavioral tests, as outlined in the experimental timeline (Fig 1A). Compared to the Con group, rats in the POP group demonstrated a marked and sustained decrease in paw withdrawal latency in the HPT at all postoperative time points, indicating persistent thermal hyperalgesia (Fig 1B). Concurrently, mechanical allodynia was observed as a significant reduction in MWT (Fig 1C). Furthermore, rotarod testing showed a decline in motor coordination (see Fig 1D for details *P* values). We assessed spontaneous pain through licking behaviors and found that the POP group exhibited a significant increase in licking frequency compared to the Con group (Fig 1E). Additionally, we evaluated motor behavior in rats using the open field test. In the POP group, rats exhibited a significant drop in moving activity (Fig 1F), as seen by reduced movement time (Fig 1G). The POP group exhibited a significant reduction in total distance traveled compared to Cons (Fig 1H), distance traveled in the central region in the center (Fig 1I), indicating pain hypersensitivity induced by surgery.

### Functional enrichment of differentially expressed genes in POP

**Fig 2:**
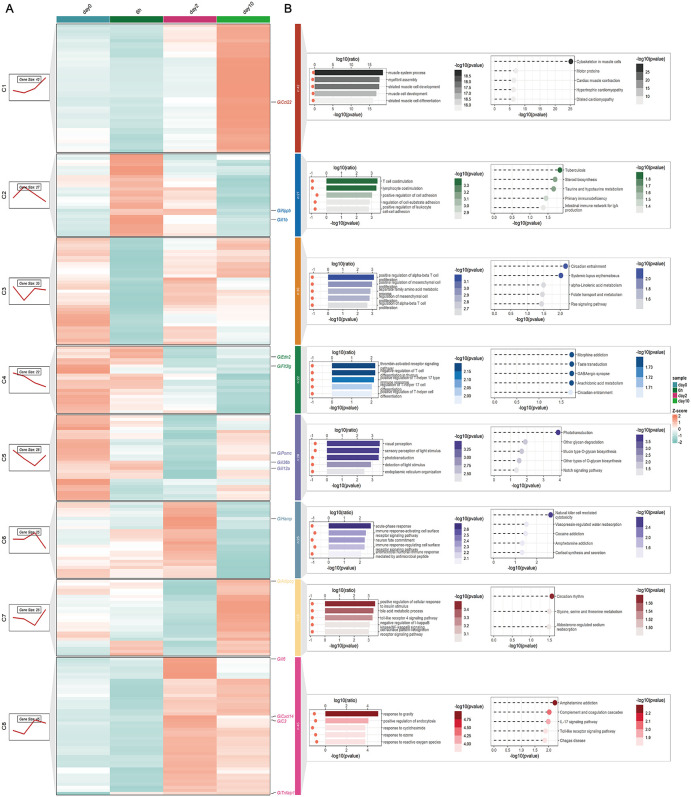
Gene expression heatmap and functional enrichment analysis of postoperative acute pain models at different time points. (A) Heatmap of acute POP-related genes. The line chart on the left side of the heatmap represents gene expression levels. The prefix “Gi” before the labeled gene names indicates a marker. (B) Corresponding group’s GO enrichment analysis and KEGG enrichment analysis.

To elucidate the molecular dynamics underlying acute POP, we performed transcriptomic profiling of DRG tissue at different time points (with full differential expression datasets detailed in S2–S7 Files). A heatmap of pain-related genes revealed distinct temporal expression patterns and a clear shift in the transcriptional landscape during pain progression (Fig 2A). Subsequent functional enrichment analysis of these dynamically regulated genes highlighted their significant involvement in biological processes and pathways critical to pain mechanisms. Specifically, GO terms were significantly enriched in inflammatory response and neuropeptide signaling pathways (Fig 2B, left). Concurrently, KEGG pathway analysis further supported these observations, showing significant enrichment of Cytokine–cytokine receptor interaction and Neuroactive ligand–receptor interaction pathways. (Fig 2B, right). Collectively, these data provide a comprehensive molecular characterization of the acute POP state, linking gene expression changes to specific cellular processes and signaling pathways. The results show that inflammatory factors are closely related to POP.

### POP elevates proinflammatory cytokines

**Fig 3:**
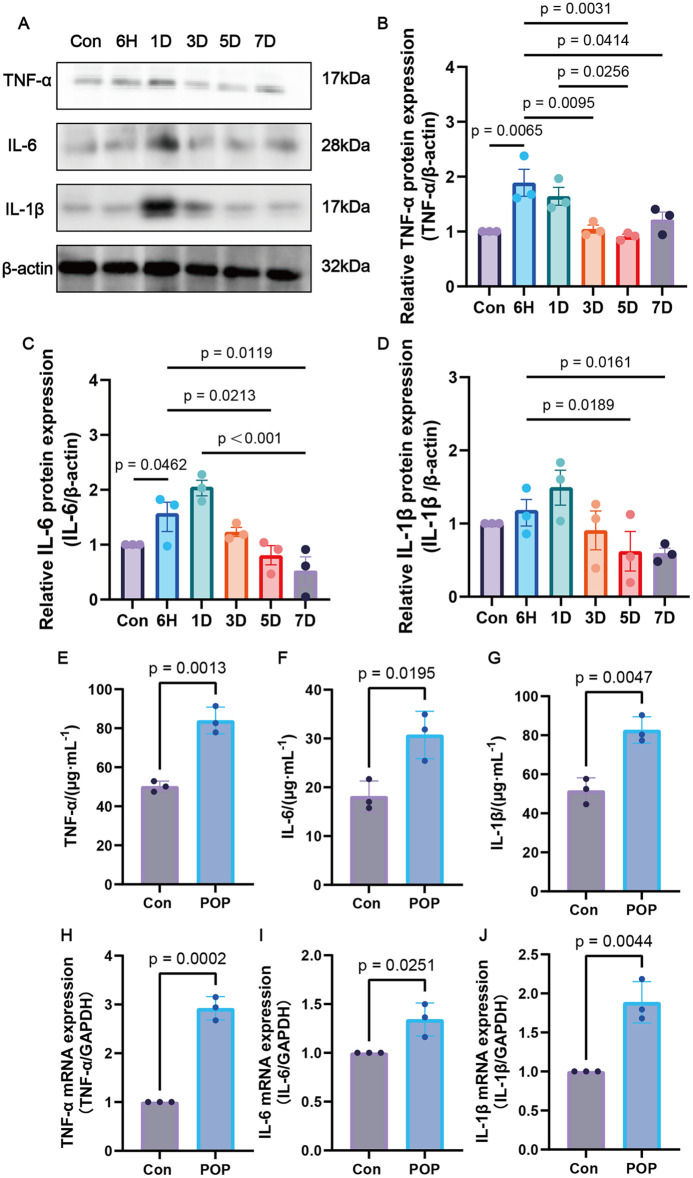
POP increases proinflammatory cytokine levels inserum and DRG. (A) Representative Western blot results: In the L4-L6 dorsal root ganglia (DRG) tissues, the protein bands of TNF-α, IL-6, IL-1β and the internal reference β-actin in the Con group (Con) and at different time points after POP induction (6H, 1D, 3D, 5D, 7D). (B-D) Quantitative analysis of Western blot: The protein expression levels of TNF-α (B), IL-6 (C) and IL-1β (D) in L4-L6 DRG (normalized with β-actin as the internal reference), showing the dynamic change trend from the Con group to different time points after POP induction. The P value represents the statistical difference compared to the Con group at each time point. (E-G) Quantitative analysis by ELISA: The concentration levels of TNF-α (E), IL-6 (F) and IL-1β (G) in the serum of rats in the Con and the POP group. (H-J) Quantitative analysis by qPCR: The mRNA expression levels of TNF-α (H), IL-6 (I) and IL-1β (J) in the L4-L6 DRG of rats in the Con and the POP group (normalized with GAPDH as the internal reference). Data were analyzed by two-way ANOVA, depending on experimental design. Non-significant results are indicated as “n. s.” Results are expressed as mean ± SEM *P* < 0.05 was considered statistically significant. n = 3.

To determine the temporal profile of neuroinflammation following surgery, we first examined the protein expression of proinflammatory cytokines in the L4–L6 DRG at multiple time points (Fig 3A and S8 File). The results demonstrated that TNF-α protein expression rapidly increased, reaching a peak at 6 h post-POP, while IL-6 and IL-1β levels peaked at 1 d (Fig 3B–D). These elevated levels were transient, returning to near-baseline values by day 3 or 5 (Fig 3B–D).

Based on these findings, we further analyzed the cytokine profiles at the 1-Day peak. In the serum, ELISA analysis revealed that the systemic levels of these cytokines were markedly elevated in the POP group (Fig 3E–G). Concurrently, in the L4–L6 DRG, the POP group exhibited significantly higher mRNA levels of TNF-α, IL-6, and IL-1β compared with the Con group (Fig 3H–J), confirming a robust local and systemic inflammatory response during the acute phase of POP

### Pro-inflammatory cytokines regulate Nav1.8 channel function and expression in DRG neurons

**Fig 4:**
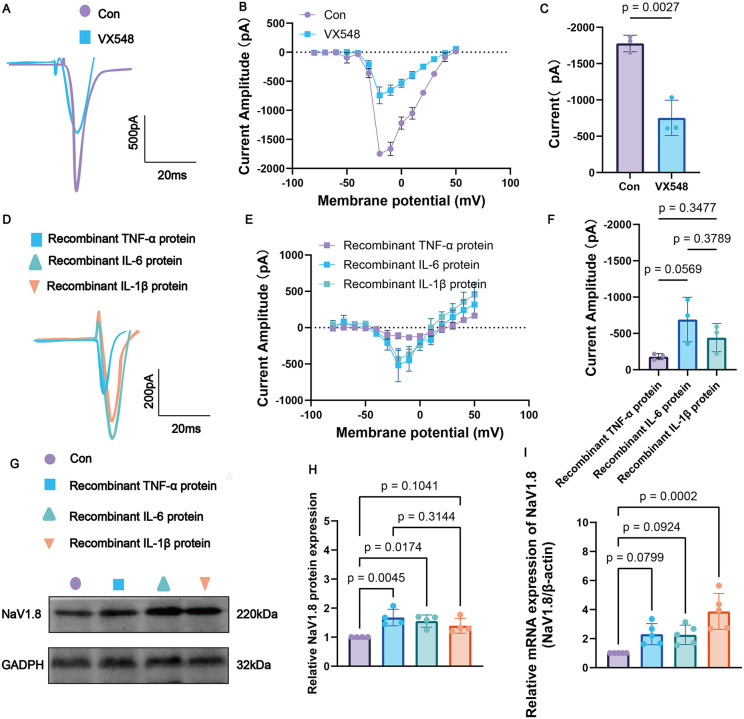
Pro-inflammatory cytokines modulate Nav1.8 channel function and expression in Rat L4-L6 DRG cells. (A) Representative current trajectories: The original recordings of Nav1.8 currents in the Con group and the Nav1.8-specific inhibitor VX548 treatment group (Con + VX548). (B) Current-voltage (I-V) relationship curves: The voltage-dependent activation curves of Nav1.8 currents in the Con group (C-BoNT/A) and the VX548 treatment group. (C) Statistical bar graphs: Comparison of the peak amplitudes of Nav1.8 currents under test potentials between the Con group and the VX548 treatment group (p = 0.0027). (D) Representative current trajectories: The original recordings of Nav1.8 currents in the Con surgery group and the recombinant pro-inflammatory factor (TNF-α, IL-6, IL-1β) treatment group. (E) Current-voltage (I-V) relationship curves: The voltage-dependent activation curves of Nav1.8 currents in the Con surgery group and each recombinant pro-inflammatory factor treatment group. (F) Statistical bar graphs: Comparison of the peak amplitudes of Nav1.8 currents between the Con surgery group and each recombinant pro-inflammatory factor treatment group (p values are marked in the figure). (G) Representative Western blot results: The bands of Nav1.8 protein and the internal reference GAPDH in the Con surgery group and each recombinant pro-inflammatory factor treatment group. (H) Western blot quantitative analysis: The relative expression levels of Nav1.8 protein (normalized with GAPDH as the internal reference), and the *P* values for comparisons between groups are marked in the figure. (I) qPCR quantitative analysis: The relative expression levels of Nav1.8 mRNA (normalized with β-actin as the internal reference), and the p values for comparisons between groups are marked in the figure. Data were analyzed using two-way ANOVA. Non-significant results were indicated as “ns”. Data were expressed as mean ± SEM. *P* < 0.05 was considered statistically significant. n = 3-5.

To explore the impact of pro-inflammatory cytokines on Nav1.8, we performed patch-clamp recordings on acutely isolated L4–L6 DRG neurons. We first validated the isolation of Nav1.8 currents using the specific inhibitor VX548 (100 μM), which significantly reduced the sodium current amplitude (Fig 4A–C). Subsequently, we observed that treatment with recombinant TNF-α, IL-6, and IL-1β modulated Nav1.8 current dynamics (Fig 4D–E). Interestingly, while protein and mRNA levels of Nav1.8 were significantly upregulated by TNF-α and IL-6 (Fig 4G–I and S8 File), the acute effect on current amplitude showed a trend toward alteration (Fig 4F).

### Impact of TNF-α on Nav1.8 channel expression and function

#### Exogenous TNF-α exacerbates thermal hyperalgesia and neuronal excitability.

**Fig 5:**
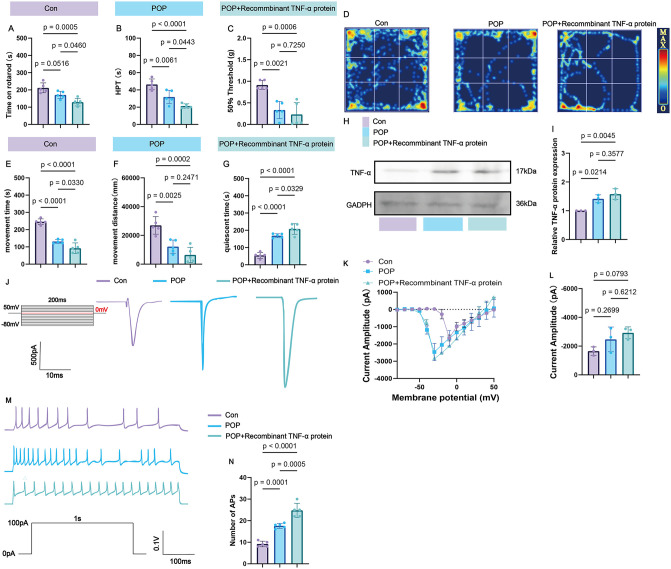
Impact of TNF-α on Nav1.8 channel activity and related nocifensive behaviors. A) Rotarod test: To evaluate the motor coordination ability of rats in the Con group, the POP model group (POP), and the POP + recombinant TNF-α protein treatment group (POP + Recombinant TNF-α protein), the time the rats stayed on the rotating rod (Time on rotarod, s) was used as the indicator. (B) Hot plate test (HPT): To assess the thermal pain hypersensitivity of each group of rats, the latency of the paw licking/ raising reaction on the hot plate (HPT, s) was used as the indicator. (C) Von Frey filament test: To evaluate the mechanical pain threshold of each group of rats, the 50% threshold of the contraction response (50% Threshold, g) was used as the indicator. (D) Representative heat map of the open field test: To show the distribution of the activity trajectories of each group of rats in the open field (the warmer the color, the more time/ activity). (E-G) Quantitative analysis of the open field test: The total movement time (E), total movement distance (F), and quiescent time (G) of each group of rats. (H) Representative band of Western blot (WB): The expression bands of TNF-α protein and internal reference GAPDH in the DRG tissues of each group of rats. (I) WB gray-scale quantitative analysis: The relative expression level of TNF-α protein in DRG of each group (standardized with GAPDH as the internal reference). (J) Representative trajectory of voltage-gated sodium current: The original records of sodium current of DRG neurons in the Con group, the POP model group, and the POP + recombinant TNF-α protein treatment group. (K) Current – voltage (I-V) relationship curve: The voltage-dependent activation curves of sodium current of DRG neurons in each group. (L) Statistical analysis of sodium current peak amplitude: The comparison of sodium current peak values of DRG neurons in each group under the test potential. (M-N) Action potential (AP) firing frequency analysis: (M) Representative action potential firing trajectory; (N) The frequency statistics of action potential firing of DRG neurons in each group under the given stimulus.Data are presented as mean ± standard error (mean ± SEM), and the comparison between groups was conducted using one-way analysis of variance (ANOVA). A difference was considered statistically significant when P < 0.05. The sample size was n = 3–5.

To further confirm the role of TNF-α in POP, we intrathecally (*i.t.*) administered recombinant TNF-α protein 30 min before surgery. Behavioral assessments revealed that exogenous TNF-α further exacerbated motor coordination deficits (Fig 5A) and thermal hyperalgesia (Fig 5B) compared to the POP group. Interestingly, while the mechanical threshold was significantly reduced in POP rats, supplemental TNF-α did not lead to a further significant decrease (Fig 5C). In the open field test, TNF-α treatment further reduced movement time (Fig 5E) and increased quiescent time (Fig 5D, G), though the total movement distance showed only a downward trend without statistical significance (Fig 5F). At the protein level, exogenous administration expectedly enhanced local expression (Fig 5H, I and S8 File). At the cellular level, although the increase in Nav1.8 current amplitude following TNF-α treatment did not reach statistical significance (Fig 5J–L), it profoundly enhanced the firing frequency of action potentials (Fig 5M–N). These data suggest that elevated TNF-α levels primarily drive POP by increasing the intrinsic excitability of L4–L6 DRG neurons.

### Behavioral and locomotor responses to POP are modulated by NF-κB inhibitor BAY 11–7082

**Fig 6:**
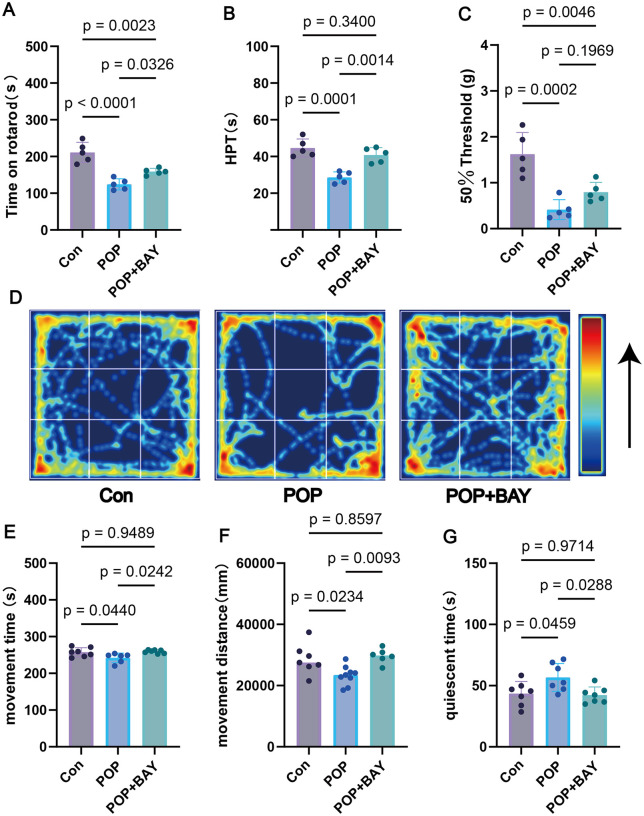
Effects of POP and NF-κB inhibitor BAY 11-7082 on pain-related behavioral and locomotor parameters in rats. (A) Rotarod test: The time spent by rats in the rotarod (Time on rotarod, s) in the Con group, the POP model group (POP), and the POP + BAY treatment group (POP + BAY) was used to evaluate the motor coordination ability. (B) Hot plate test (HPT): The latency of thermal pain response (HPT, s) of rats in each group was used to assess thermal pain hypersensitivity. (C) Von Frey filament test: The 50% threshold of paw withdrawal response (50% Threshold, g) of rats in each group was used to evaluate mechanical pain hypersensitivity. (D) Representative heat map of the open field test: It shows the distribution of the activity trajectories of rats in the open field (the warmer the color, the more time or activity). (E-G) Quantitative analysis of the open field test: The total movement time (E, Movement time, s), total movement distance (F, Movement distance, mm), and quiescent time (G, Quiescent time, s) of rats in each group.The data are presented as mean ± standard error (mean ± SEM), and the comparison between groups was conducted using two-way analysis of variance (two-way ANOVA). n = 5-9.

To evaluate the effects of the NF-κB inhibitor BAY 11–7082 on pain-related behaviors and locomotor activity, we performed a series of behavioral assessments. Rats were divided into three groups: Con, POP, and POP + BAY (POP rats treated with the NF-κB inhibitor BAY 11–7082). In pain-related behavioral tests, the POP group exhibited a significant decrease in rotarod latency (*P* < 0.0001, Fig 6A) and HPT latency (*P* < 0.0001,Fig 6B), along with a marked reduction in the 50% withdrawal threshold (*P* = 0.0002, Fig 6C). Notably, our data reveal a functional specificity within the investigated regulatory pathway. While the TNF-α/NF-κB/Nav1.8 axis significantly contributes to the development of thermal and spontaneous pain, its impact on mechanical allodynia was found to be limited (*P* = 0.1969, POP vs. POP + BAY, Fig 6C). This suggests that in the plantar incision model, distinct molecular mechanisms or alternative signaling cascades may independently mediate different modalities of nociceptive hypersensitivity.

For locomotor activity, heatmap representations demonstrated altered movement patterns in the POP group (Fig 6D), which were restored by BAY 11–7082 administration. Quantitative analysis showed that POP significantly decreased movement time (*P* = 0.0440) and movement distance (*P* = 0.0234), while increasing quiescent time (*P* = 0.0459) compared to the Con group (Fig 6E–G). BAY 11–7082 intervention attenuated these locomotor alterations, with *P* = 0.0242 for movement time, *P* = 0.0093 and reversing the elevation in quiescent time (*P* = 0.0288; Fig 6E–G).

### Intrathecal injection of the NF-κB inhibitor BAY 11–7082 modulates Nav1.8

**Fig 7:**
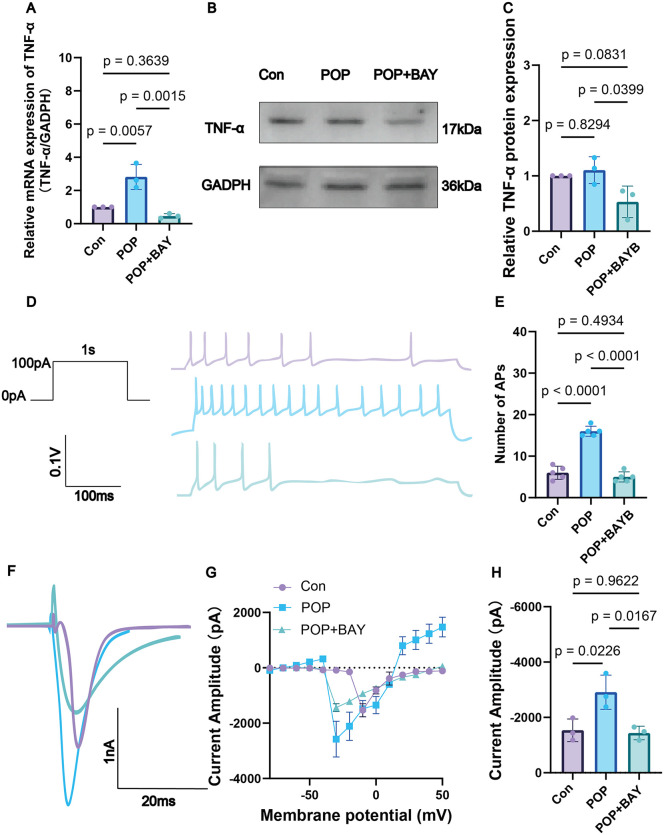
Effects of POP and intrathecal injection of NF-κB inhibitor BAY on inflammatory cytokine expression and electrophysiological properties in rats. (A) qPCR quantitative analysis: The relative mRNA expression levels of TNF-α in L4-L6 DRG of rats in the Con, the POP group, and the POP + BAY treatment group (POP + BAY) were normalized using GAPDH as the internal reference. (B) Representative Western blot results: The expression bands of TNF-α protein (17 kDa) and the internal reference GAPDH (36 kDa) in DRG tissues of each group. (C) Western blot gray-scale quantitative analysis: The relative expression levels of TNF-α protein in DRG of each group (normalized using GAPDH as the internal reference). (D) Representative action potential firing trajectories: The original records of action potential firing of DRG neurons in each group under 100 pA current stimulation. (E) Comparison of action potential firing frequency: The number of action potential firing of DRG neurons in each group under 100 pA stimulation. (F) Representative voltage-gated sodium current trajectories: The original records of sodium current of DRG neurons in each group. (G) Current-voltage (I-V) relationship curves: The voltage-dependent activation curves of sodium current of DRG neurons in each group. (H) Statistical comparison of sodium current peak amplitude: The comparison of sodium current peak values of DRG neurons in each group under the test potential.The data are presented as mean ± standard error (mean ± SEM), and the comparison between groups was conducted using two-way analysis of variance (two-way ANOVA). A P value less than 0.05 is considered to indicate a statistically significant difference. The P value is indicated in the figure. The sample size is n = 3–5.

To investigate the regulatory role of the TNF-α/NF-κB axis in POP, we assessed the expression of inflammatory cytokines and Nav1.8-mediated electrophysiological properties following intrathecal administration of BAY 11–7082.. At the mRNA level, qPCR analysis (Fig 7A) showed that POP significantly increased the expression of TNF-α (*P* = 0.0057 vs. Con), while BAY 11–7082 treatment effectively reversed this elevation (*P* = 0.0015 vs. POP). Consistently, Western blot analysis demonstrated that BAY 11–7082 significantly reduced TNF-α protein levels compared to the POP group (*P* = 0.0399; Fig 7B–C, and S8 File).

Regarding Nav1.8-mediated electrophysiological properties, representative recordings of action potentials and Nav1.8-mediated sodium currents (Fig 7D–E) along with current-voltage analysis (Fig 7F–H) revealed that intrathecal BAY 11–7082 effectively restored Nav1.8 sodium current amplitude compared to the Con group (*P* < 0.0001), suggesting that BAY 11–7082 modulates Nav1.8-dependent electrophysiological activity.

## Discussion

The present study reveals a regulatory axis in plantar POP: pro-inflammatory cytokine TNF-α may activate the NF-κB signaling pathway, which in turn upregulates Nav1.8 expression in L4-L6 DRG neurons, ultimately exacerbating nociceptive hypersensitivity and locomotor dysfunction. This mechanism is consistent with and extends previous research on pain signaling networks.

First, our observation that POP induces spinal NF-κB activation and subsequent inflammatory cytokine upregulation aligns with previous studies demonstrating that plantar incision triggers NF-κB p65 overexpression in the spinal dorsal horn as early as 30 minutes post-surgery, with sustained elevation for 3 days [22]. The attenuation of thermal hyperalgesia (and partial improvement in locomotor activity) by intrathecal NF-κB inhibitor BAY 11–7082 in the present study further validates NF-κB as a pivotal mediator of incisional pain—mirroring findings that intrathecal PDTC (another NF-κB inhibitor) mitigates incision-induced hypersensitivity in the plantar incision model [22], and nanotherapy targeting NF-κB p65 reduces acute pain after joint injury [23]. These consistencies suggest the evolutionary conservation of NF-κB-dependent pain pathways across rodent models of surgical injury. In addition, Bay 11–7082 has been reported to relieve pain behaviors in neuropathic pain models [24].

Second, our focus on Nav1.8 aligns with its well-established role in nociception. A study demonstrated that VX548-sensitive Nav1.8 currents in DRG neurons are altered in incisional pain models, supporting our finding that Nav1.8 function (rather than mere expression) is critical for pain pathogenesis. Moreover, the selective Nav1.8 antagonist A-803467 has been shown to attenuate inflammatory and neuropathic pain, further supporting the importance of Nav1.8 in pain signaling [25]. Electrophysiological data showing BAY 11–7082 reduces Nav1.8 current amplitude further echoes reports that spinal cord injury enhances Nav1.8-mediated transient (I_NaT) and resurgent (I_NaR) sodium currents to increase DRG neuron excitability, underscoring Nav1.8 as a downstream effector of pain signaling [26].

This study clarifies two key mechanistic links: Notably, although carprofen (5 mg/kg) was administered for animal welfare, significant pain behaviors and molecular upregulation persisted, suggesting that the TNF-α/NF-κB axis represents a “breakthrough” mechanism that is not fully suppressed by conventional NSAIDs at this dose. Then, TNF-α-driven NF-κB activation in POP, and NF-κB-dependent Nav1.8 regulation. Notably, our findings are highly consistent with recent work by Lima et al. (2022), who demonstrated that the TNF-α/NF-κB pathway modulates Nav1.8 expression in a similar model of incisional pain [27]. While Lima et al. highlighted the role of this axis in mechanical hyperalgesia, our data extend these findings by providing direct electrophysiological evidence. We showed that recombinant TNF-α not only increased Nav1.8 mRNA and protein levels but also significantly altered its current properties in DRG neurons—effects that were effectively abolished by the NF-κB inhibitor BAY 11–7082. This convergence of evidence across different studies reinforces the hypothesis that the TNF-α/NF-κB/Nav1.8 axis is a fundamental driver of postoperative pain pathogenesis.

Notably, we observed discordance between Nav1.8 expression and function: cytokine treatment increased Nav1.8 protein levels but decreased current amplitude, which may reflect post-translational modifications (e.g., phosphorylation) or altered channel trafficking—processes known to regulate Nav1.8 gating [28]. This complexity highlights that NF-κBcontrols Nav1.8 via multiple layers, extending beyond transcriptional upregulation [29]. The convergence of behavioral (restored paw threshold, locomotor activity) and molecular (reduced cytokines/Nav1.8) data confirms that targeting this axis reverses POP pathophysiology at both functional and structural levels.

The identification of the TNF-α/NF-κB/Nav1.8 axis offers translational value for POP management. Current analgesics (e.g., opioids) carry risks of dependence, while Nav1.8 antagonists have shown promise in preclinical models but lack specificity. Our results suggest a dual-target strategy: inhibiting NF-κB (e.g., via intrathecal BAY 11–7082) could simultaneously reduce inflammation and normalize Nav1.8 function, avoiding off-target effects of direct Nav1.8 blockers. Intrathecal delivery of BAY 11–7082 is particularly relevant, as spinal NF-κB activation is a proximal event in POP, and this route minimizes systemic toxicity. Clinical precedent for NF-κB-targeted analgesia exists (e.g., glucocorticoids modulate NF-κB in joint surgery), but BAY 11–7082’s specificity for NF-κB makes it a more precise tool.

This study has several limitations. First, we focused on TNF-α, but IL-1β and IL-6 also modulated Nav1.8—future work should investigate whether these cytokines act via parallel NF-κB pathways or alternative cascades. Second, electrophysiological recordings were limited to acute DRG cultures, and our assessments utilized a relatively limited sample size (n = 3–5 neurons) and a single current injection intensity (100 pA). While these data provide consistent functional trends, future studies with larger cohorts and multi-intensity step protocols will be needed to fully characterize the recruitment properties of Nav1.8; in vivo patch-clamp studies would better reflect physiological Nav1.8 activity in POP. Third, we did not assess NF-κB binding to the Nav1.8 promoter, which is needed to confirm direct transcriptional regulation. Furthermore, it is noteworthy that at the 6-hour peak, rats exhibited a transient decrease in motor coordination, as indicated by the Rotarod test. This could potentially act as a confounding factor for evoked pain assessments like the Von Frey and Hot plate tests. However, the presence of vigorous spontaneous licking and the rapid nature of the withdrawal reflexes suggest that the primary driver of the observed behaviors was mechanical and thermal hyperalgesia rather than pure motor deficit.

Future research could address these gaps by: (1) performing chromatin immunoprecipitation (ChIP) to validate NF-κB-p65 binding to *SCN10A*; (2) using conditional NF-κB knockout rat to dissect DRG-specific vs. spinal contributions; (3) testing combination therapy with TNF-α neutralizing antibodies and BAY 11–7082 for synergistic analgesia. Additionally, exploring long-term effects of NF-κB inhibition on CPSP development would enhance clinical relevance.

In summary, our findings indicate that TNF-α promotes the NF-κB signaling pathway to upregulate Nav1.8 expression and disrupt its electrophysiological function in DRG neurons, thereby exacerbating plantar POP and locomotor dysfunction. Intrathecal NF-κB inhibitor BAY 11–7082 reverses these pathological changes by targeting this axis. This work not only advances our understanding of POP pathogenesis but also provides a rationale for developing NF-κB-targeted therapies to improve POP management.

## Supporting information

S1 File Primer sequences used for real-time quantitative PCR (RT-qPCR). This table lists the exact 5’ to 3’ nucleotide sequences of the forward and reverse primers utilized for amplifying targeting genes (IL-6, IL-1β, TNF-α, Nav1.8) and the reference gene (GAPDH).
(DOCX)

S2 File Differential expression analysis results (6h vs. Day 0). This dataset contains the full DESeq2 output comparing the gene expression profiles between the 6-hour postoperative group and the day 0 baseline group.
(TXT)

S3 File Differential expression analysis results (Day 2 vs. 6h). This dataset contains the full DESeq2 output comparing the gene expression profiles between the day 2 postoperative group and the 6-hour postoperative group.
(TXT)

S4 File Differential expression analysis results (Day 2 vs. Day 0). This dataset contains the full DESeq2 output comparing the gene expression profiles between the day 2 postoperative group and the day 0 baseline group.
(TXT)

S5 File Differential expression analysis results (Day 10 vs. 6h). This dataset contains the full DESeq2 output comparing the gene expression profiles between the day 10 postoperative group and the 6-hour postoperative group.
(TXT)

S6 File Differential expression analysis results (Day 10 vs. Day 0). This dataset contains the full DESeq2 output comparing the gene expression profiles between the day 10 postoperative group and the day 0 baseline group.
(TXT)

S7 File Differential expression analysis results (Day 10 vs. Day 2). This dataset contains the full DESeq2 output comparing the gene expression profiles between the day 10 postoperative group and the day 2 postoperative group.
(TXT)

S8 File Original Western blot images. This file contains the uncropped and unedited full-length gel and blot images used for protein expression analysis in the study.
(PPT)

S9 File Graphical abstract. (PNG)
