# Supplementary material for: TNF-α exacerbates postoperative plantar pain by regulating the expression of Nav1.8
Source: PLoS One. 2026 Jul 17;21(7):e0351249. doi: 10.1371/journal.pone.0351249 (PMC13379010; doi:10.1371/journal.pone.0351249)
Supplement: S1 File — This table lists the exact 5’ to 3’ nucleotide sequences of the forward and reverse primers utilized for amplifying targeting genes (IL-6, IL-1β, TNF-α, Nav1.8) and the reference gene (GAPDH). (DOCX) [file pone.0351249.s001.docx]

S1 File. Primer sequences used for real-time quantitative PCR

| Gene | Forward primer (5′→3′) | Reverse primer (5′→3′) |
| --- | --- | --- |
| IL-6 | GTAGCCGCCCCACACAGA | CATGTCTCCTTTCTCAGGGCTG |
| IL-1β | TGTTTCCCTCCCTGCCTCTGAC | CGACAATGCTGCCTCGTGACC |
| TNF-α | CACCCCCTCCCTATCTCTCA | CCCTACACCCTTCTCACTCGAA |
| Nav1.8 | ACCGACAATCAGAGCGAGGAG | ACAGACTAGAAATGGACAGAATCACC |
| GAPDH | AAGTTCAACGGCACAGTCAAGG | GACATACTCAGCACCAGCATCAC |
