# Supplementary figures and images for: TNF-α exacerbates postoperative plantar pain by regulating the expression of Nav1.8
Source: PLoS One. 2026 Jul 17;21(7):e0351249. doi: 10.1371/journal.pone.0351249 (PMC13379010; doi:10.1371/journal.pone.0351249)

## Slide 1
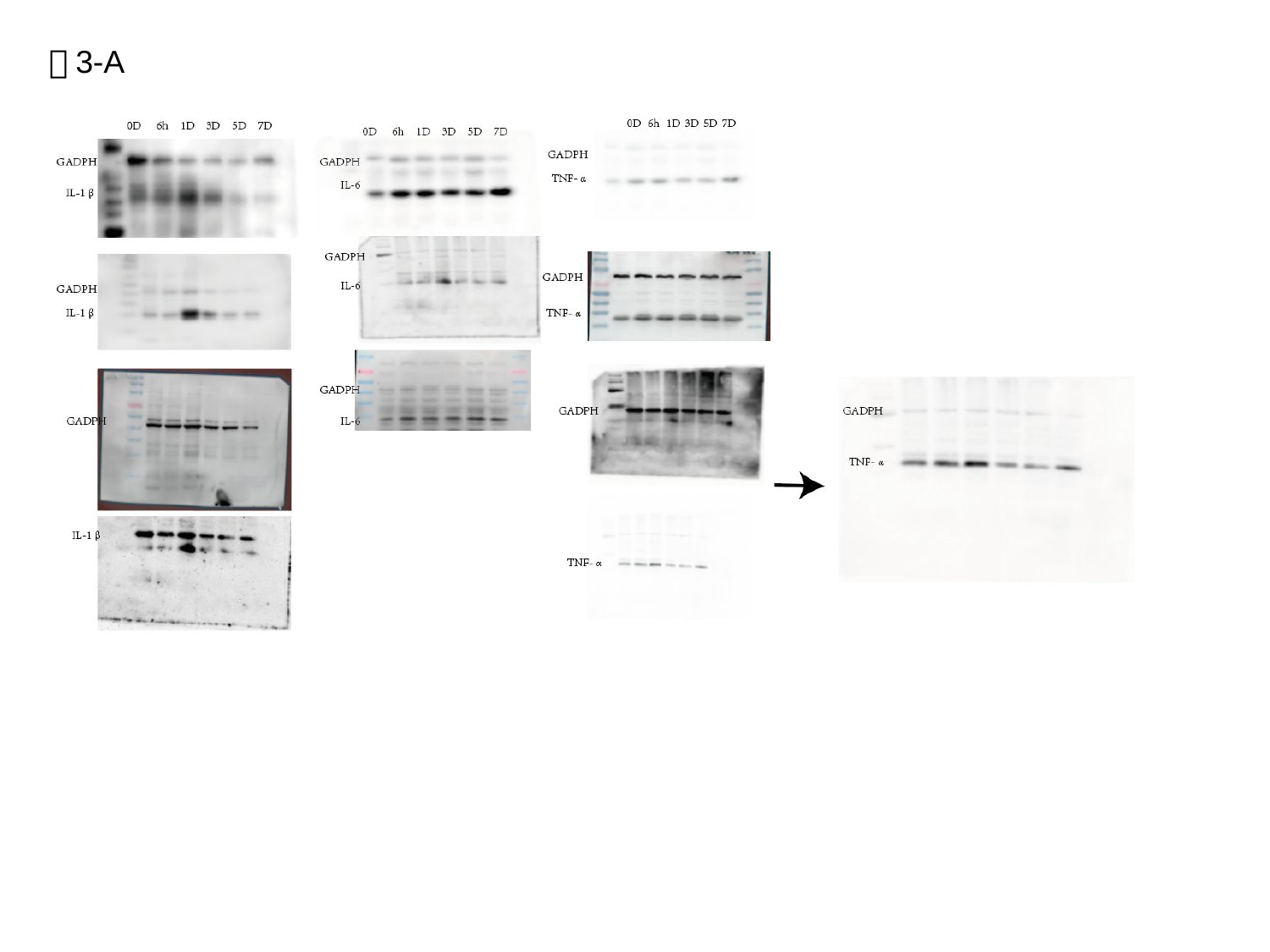

图3-A

## Slide 2
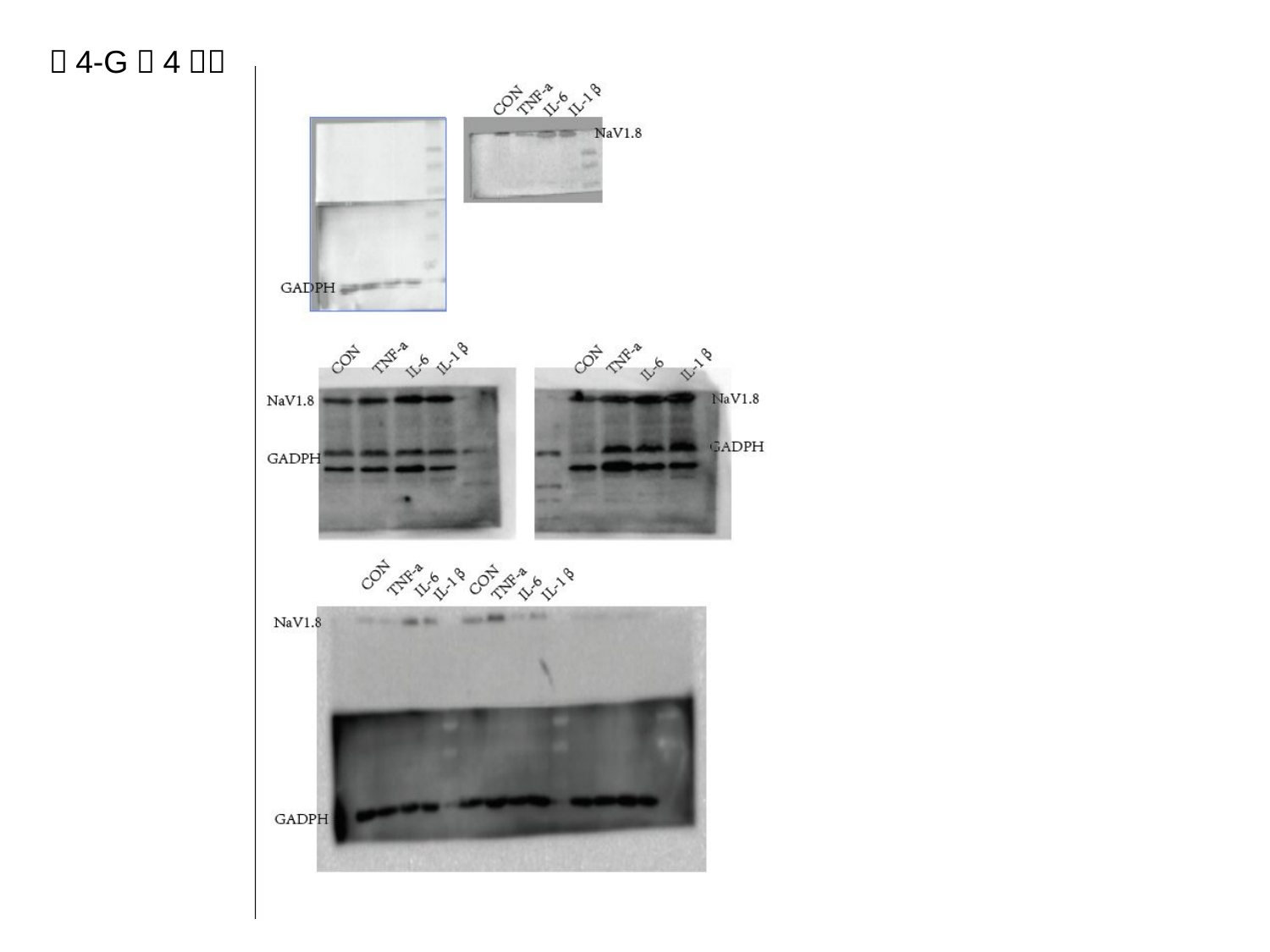

图4-G（4组）

## Slide 3
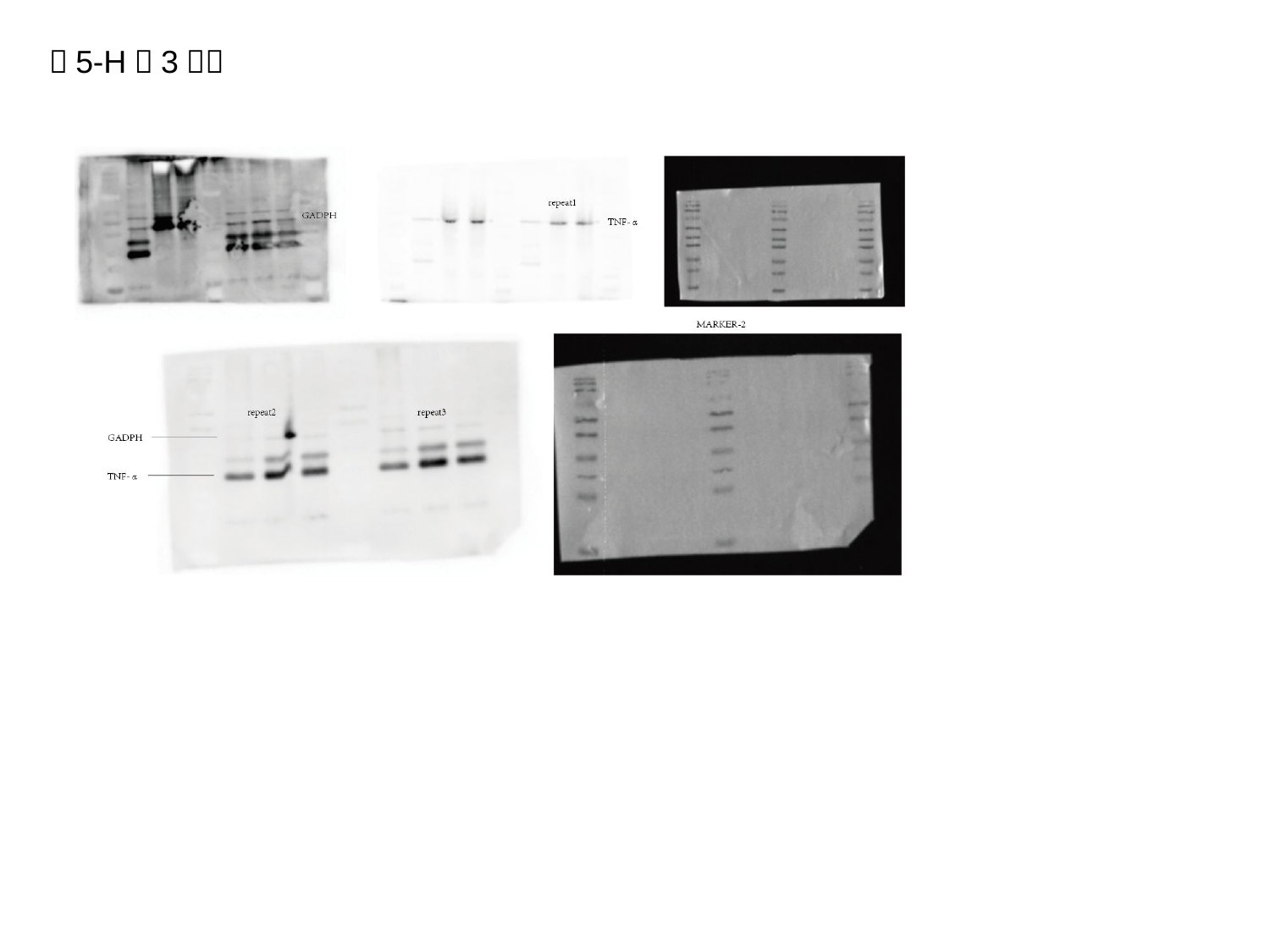

图5-H（3组）

## Slide 4
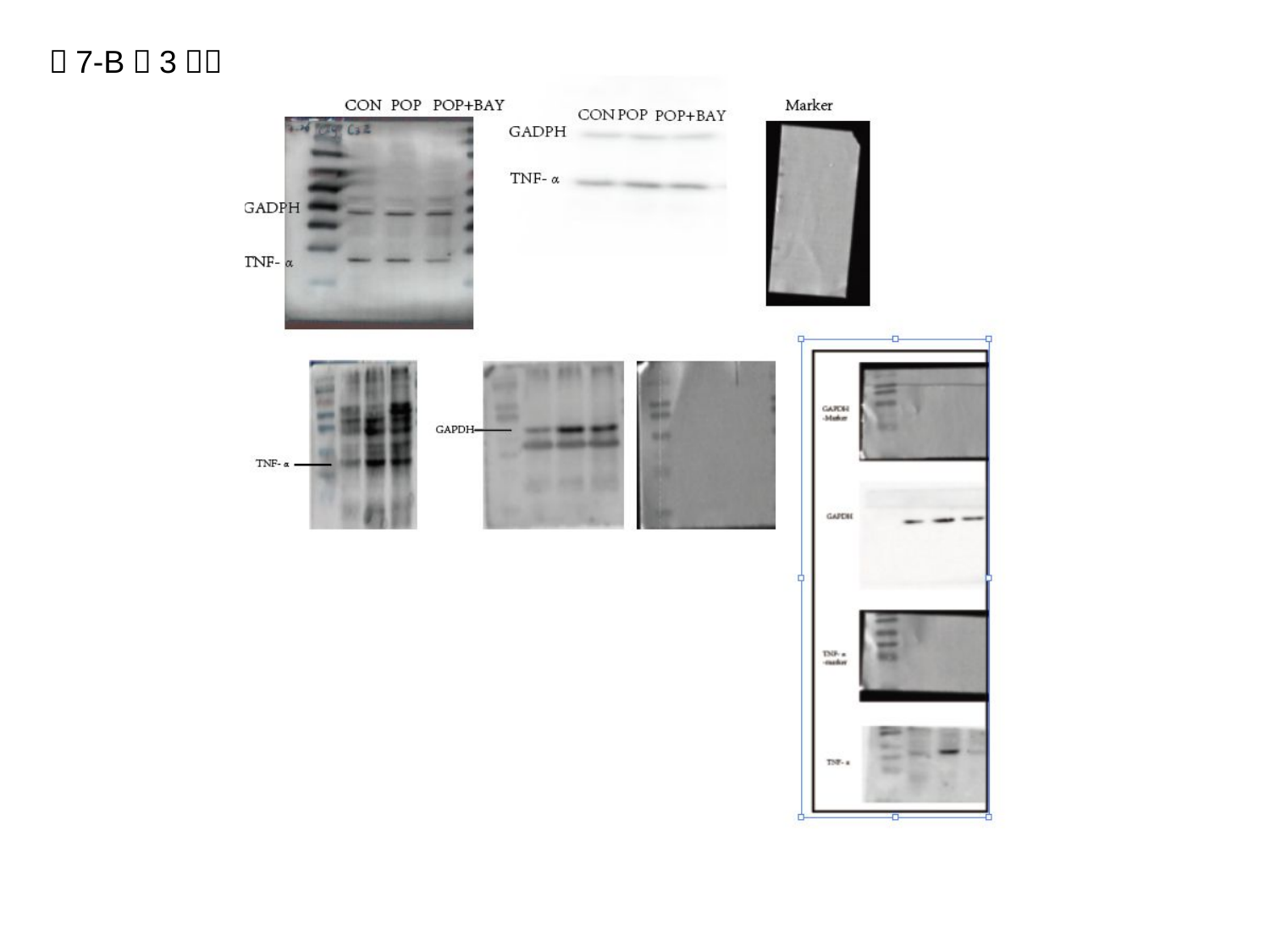

图7-B（3组）

Supplement: S8 File — This file contains the uncropped and unedited full-length gel and blot images used for protein expression analysis in the study. (PPT) [file pone.0351249.s008.ppt]

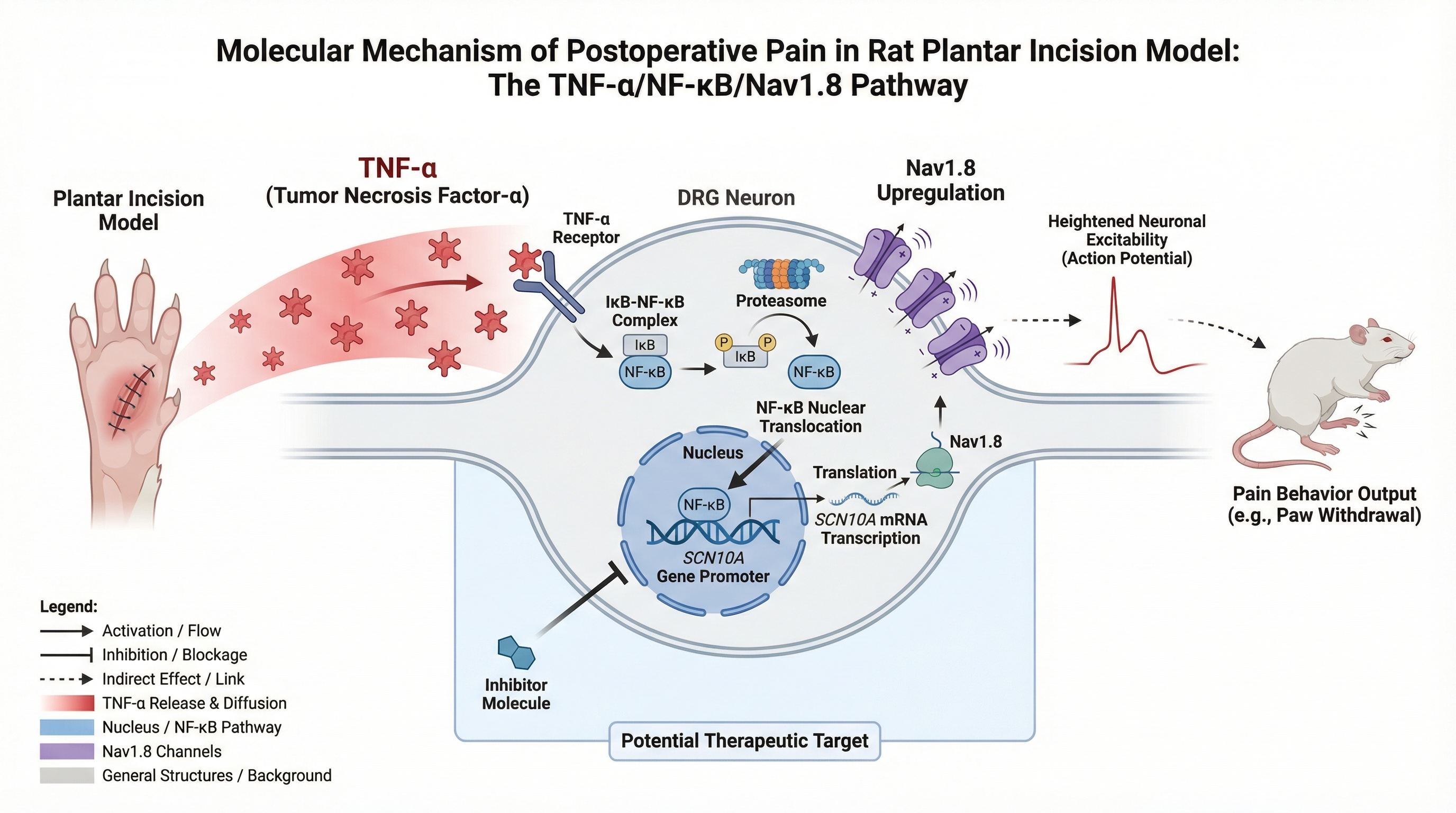

Supplement: S9 File — (PNG) [file pone.0351249.s009.png]
